# MicroRNA Microarray Profiling in Infantile Hemangiomas

**Published:** 2019-04-16

**Authors:** Brent Earl Schultz, Christopher R. Spock, Laura K. Tom, Yong Kong, Karina T. Canadas, Samuel Kim, Milton Waner, Teresa O., Richard Antaya, Deepak Narayan

**Affiliations:** ^a^Yale School of Medicine, New Haven, Conn; ^b^Yale School of Public Health: Biostatistics, New Haven, Conn; ^c^Section of Otolaryngology; ^d^Section of Plastic and Reconstructive Surgery; ^e^Departments of Dermatology and Pediatrics, Yale School of Medicine, New Haven, Conn; ^f^Vascular Birthmark Institute, New York, NY

**Keywords:** infantile hemangioma, microRNA, miRNA, Piwi-interacting RNA, microarray profiling

## Abstract

**Objective:** MicroRNAs are short, noncoding RNA molecules that negatively regulate the stability and translational efficiency of target mRNAs. They are critical regulators of growth and development. Our aim was to identify microRNAs involved in the growth and regulation of infantile hemangiomas. In addition, we searched for the presence of Piwi-interacting RNAs in hemangioma tissue as another regulator of infantile hemangiomas. **Methods:** RNA was extracted from hemangioma specimens from 3 clinical, age-based categories: proliferative (N = 16), quiescent (N = 8), and involuting (N = 9). RNAs from human dermal microvascular endothelial cells were used as controls. MicroRNA microarray was performed, and the expression profiles of the hemangiomas and endothelial cells were compared using the *t* test. 5′ End-labeling of RNA of our hemangioma specimens was performed for Piwi-interacting RNA detection. **Results:** Analysis confirmed statistically significant downregulated (N = 18) and upregulated (N = 15) microRNAs. Piwi-interacting RNA analysis did not detect Piwi-interacting RNA transcripts in the hemangioma specimens. **Conclusions:** The differential expression of microRNAs found in our hemangioma specimens provides insight into the regulation of hemangioma formation and proliferation, quiescence, and fibrofatty involution. Piwi-interacting RNA transcripts were not detected in the hemangioma specimens. These novel findings will help in establishing new therapeutic and diagnostic initiatives for these tumors.

MicroRNAs (miRNAs) are the products of miRNA genes found in both plants and animals. miRNA genes are transcribed into precursor miRNAs and after several processing steps, small mature miRNA molecules are generated, typically 20 to 24 nucleotides in length. These miRNAs do not themselves code for proteins, but they regulate the production of many proteins in the cell by binding to complementary target mRNAs. This interaction leads to either mRNA degradation or inhibition of protein synthesis at the posttranscription mRNA level. The final effect is a decrease in the production of protein from genes that are regulated by the particular miRNA.^1^^-^11 In humans, miRNAs represent approximately 1% of all genes and are estimated to regulate protein production of 10% or more of proteins.^12^^,^^13^

Soon after their discovery, due to their recurring role in development and regulatory processes, these small entities were postulated to play a role in cancer.^14^^-^16 The use of miRNA microarray technology allows the levels of hundreds of miRNAs to be studied simultaneously.^17^ Comprehensive and more extensive reviews of the technical aspects and pitfalls of microarrays have been published.^18^^,^^19^ Changes in the expression of miRNAs have been observed in a variety of human tumors. Although expression differences may not necessarily reflect causal events of tumorigenesis, such changes may, nevertheless, regulate genes important in tumor pathogenesis and may be useful for classifying tumors and predicting their outcomes. Examples of such gene expression alterations in miRNAs were first detected in colorectal neoplasia^16^^,^^20^ and later in a number of cancers including chronic lymphoblastic leukemia,^21^ Burkitt lymphoma,^22^^,^^23^ B-cell lymphoma,^22^^,^^24^^-^27 pituitary tumors,^28^^,^^29^ lung cancer,^30^^,^^31^ breast cancer,^32^^-^35 ovarian cancer,^36^^,^^37^ brain tumors,^38^^-^41 papillary thyroid cancer,^42^ and hepatocellular carcinoma.^43^^,^^44^

Recently discovered Piwi-interacting RNAs (piRNAs) represent an additional class of small, noncoding RNAs distinct from miRNAs that interact with the Piwi subfamily of Piwi-Ago family proteins.^45^^-^48 Most piRNAs map to unique sites in the genome, including intergenic, intronic, and exonic sequences. For this reason, piRNAs may have diverse functions including epigenetic programming, repressing transposition, and posttranscriptional regulation supported by the known function of the partner Piwi proteins. For instance, Piwi is an epigenetic regulator^49^ and it is thus possible that Piwi-associated piRNAs may also be involved in epigenetic regulation. In addition, Piwi prevents retrotransposon transposition in the testicular germ line, which suggests a second role in Piwi-associated piRNAs.^50^

Hemangiomas are the most common benign tumor of infancy with an overall incidence between 1% and 5%.^51^^-^54 They are a well-studied tumor from a clinical standpoint. More than 50 years ago, these lesions were described clinically as being vascular lesions characterized by a period of rapid growth, usually immediately within the first months of birth, followed by involution.^55^ More recently, a biologic classification of these lesions among other vascular birthmarks was established by Mulliken and Glowacki^56^ on the basis of clinical manifestations, histopathology, and natural history.^57^ Complete involution occurs at approximately 10% per year, with 50% involuted by 5 years of age, 70% by 7 years of age, and 90% by 9 years of age.^58^ With these lesions being as clinically well-described as they are, it is interesting that their origin and pathogenesis continue to elude physicians.

Our aim in conducting a miRNA microarray analysis of hemangioma tissue in various stages of growth was to determine whether a differential expression in miRNAs existed between hemangiomas and normal endothelial cells. This would elucidate whether an involvement of miRNAs in the development and progression of hemangiomas exists. In addition, we searched for the presence of piRNAs in hemangioma tissue because of their possible role in the germ line and in maintenance of stem cell pluripotency. A unique expression pattern of piRNAs in hemangiomas would provide further insight into the origin and pathogenesis of these lesions.

## METHODS

### Specimen collection

All samples were collected in accordance with an approved HIC protocol as reviewed by the Yale University Medical School institutional review board (HIC#0507000430). As these samples were collected from children, fully informed parental and childhood assent, when age-appropriate, was obtained prior to surgery. Only the tissue remaining following collection of the pathological specimen was used for this experiment.

Specimens were separated into 3 categories: (1) proliferative, (2) quiescent, and (3) involuting phases. These categories were determined on a clinical basis, as well as by age. The ages of samples were calculated from birth to time of resection. Proliferative hemangiomas were less than 1.5 years old, with interval growth between the last 2 clinic visits proceeding surgery. Quiescent hemangiomas were older than 1 year, demonstrating no interval growth between the last 2 clinic visits preceding surgery. Involuting hemangiomas were at least 2 years old, with interval regression by measurement between the last 2 clinic visits preceding surgery.

Hemangioma samples were obtained during resection, minced in Qiagen RNA Later solution, and stored at −80°C.

Thirty-three hemangioma samples were collected with the following ages: proliferative (N = 16)—81, 81, 123, 140, 162, 164, 165, 165, 165, 216, 273, 286, 286, 299, 380, and 407 days (*x* = 212 days); quiescent (N = 8)—299, 380, 455, 480, 590, 590, 752, and 1083 days (*x* = 579 days); and involuting (N = 9)—752, 1171, 1471, 1704, 1712, 2304, 3626, 3626, and 5490 days (*x* = 2428 days). In addition, 4 normal endothelial cell control lines were analyzed at passage 4: 2 adult and 2 neonatal human dermal microvascular endothelial cells (HDMECs) (see the “Endothelial cell purification and culturing” section).

### Endothelial cell purification and culturing

This protocol was optimized at the Yale Skin Diseases Research Center, New Haven, Conn. HDMECs were isolated from normal adult skin obtained as discarded tissue from Yale-New Haven Hospital, New Haven, Conn, under an approved HIC protocol. Purified endothelial cells were plated on fibronectin-coated plastic and grown to confluence and expanded. Neonatal HDMECs, isolated from pooled foreskin, were purchased from Cambrex (East Rutherford, NJ) and grown to confluence as noted earlier. Both adult and neonatal cells at passage 3 were subjected to FACS analysis; cultures that were 90% or greater CD31 positive, a marker for endothelial cells, were RNA extracted at passage 4.

### RNA extraction

Following liquid nitrogen powder homogenization of each sample, total RNA isolation was done using TRIzol (Invitrogen, Carlsbad, Calif) according to the manufacturer's specifications with the following exceptions. Once the initial phase separation was accomplished with the addition of phenol/chloroform, the samples were vigorously vortexed to shear genomic DNA to ensure an uncontaminated RNA sample separation. The RNA phase was then subjected to a second 1:24 isoamyl alcohol/chloroform extraction to minimize potential phenol contamination that could inhibit downstream enzymatic applications. To remove potential genomic contamination, 10 μg of total RNA from each sample was then treated with DNase Qiagen mini-elute columns according to the manufacturer's specifications. RNA integrity was then assessed using 1 μL of sample on the Agilent (Santa Clara, CA) bioanalyzer 2100 (provided as a service of the Keck Center at Yale University). Band intensities of 18S and 28S RNA were quantified, and samples with an 18S/28S ratio of 1.8 or greater were utilized. Following quality control, each sample was converted into cDNA using the ABI 4368813 cDNA archive kit. All samples were stored at −80°C.

### miRNA microarray analysis*

The assay started with 2 to 5 µg of total RNA sample, which was size fractionated using a YM-100 Microcon centrifugal filter (from Millipore, Burlington, MA) and the small RNAs (<300 nucleotides) isolated were 3′-extended with a poly(A) tail using poly(A) polymerase. An oligonucleotide tag was then ligated to the poly(A) tail for later fluorescent dye staining; 2 different tags were used for the 2 RNA samples in dual-sample experiments.

On the microfluidic chip, each detection probe consisted of a chemically modified nucleotide coding segment complementary to target miRNA (from miRBase, 11.0; http://microrna.sanger.ac.uk/sequences) and a spacer segment of polyethylene glycol to extend the coding segment away from the substrate. The detection probes were made by in situ synthesis using PGR (photogenerated reagent) chemistry. Hybridization was performed overnight on a µParaflo microfluidic chip using a micro-circulation pump (Atactic Technologies, Houston, TX). The hybridization melting temperatures were balanced by chemical modifications of the detection probes. Hybridization used 100 µL 6xSSPE buffer (0.90 M NaCl, 60 mM Na_2_HPO_4_, 6 mM EDTA, pH 6.8) containing 25% formamide at 34°C. After hybridization, detection used fluorescence labeling, using tag-specific Cy3 and Cy5 dyes. Hybridization images were collected using a laser scanner (GenePix 4000B; Molecular Device, San Jose, CA) and digitized using Array-Pro image analysis software (Media Cybernetics, Rockville, MD). Data were analyzed by first subtracting the background and then normalizing the signals using a LOWESS filter (locally weighted regression). Each probe was included on the chip 7 times, and from these signals an average and standard deviation were calculated.

The hemangioma cases were compared with the 4 controls using a 2-sample, 2-tailed *t* test, assuming unequal variances. The *P* values were ranked and compared with the Bonferroni correction for multiple comparisons. Specifically, the *P* values needed to attain a level of significance were determined by the following calculation: 0.05/886 = .000056, where 886 equaled the number of markers examined.

### 5′ End-labeling of RNA for piRNA detection

5′ End-labeling of RNA of our hemangioma specimens was performed for piRNA detection according to industry standards.

## RESULTS

### miRNA microarray analysis

Data analysis generated a list of miRNAs expressed in hemangiomas at a level statistically different, using *P* < .000056, from the control adult and neonatal HDMECs (Table 1). A total of 18 downregulated miRNAs and 15 upregulated miRNAs were statistically significant.

### piRNA analysis

**Figure 1:**
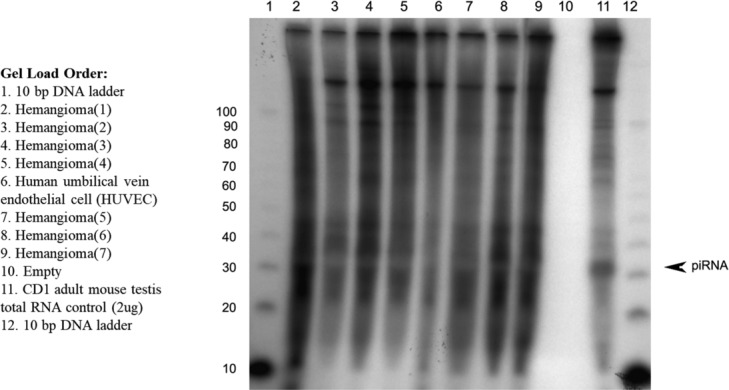
piRNA expression in hemangiomas. This figure depicts the absence of a piRNA band in all 7 hemangioma specimens as well as the control sample of human umbilical vein endothelial cells after running labeled RNA on polyacrylamide gel.

We selected 7 hemangioma specimens and 1 control sample of human umbilical vein endothelial cells to screen for the presence of piRNAs. piRNA analysis did not detect piRNA transcripts in the hemangioma specimens (Fig 1).

## DISCUSSION

Since the conception of miRNAs in 1993 as small RNAs that regulate developmental transitions in worm larvae (*Caenorhabditis elegans)*,^8^^-^11^,^^59^ considerable research was conducted to decipher the role of these molecules. Ensuing studies demonstrated tissue-specific and developmental stage-specific miRNA expression as well as conservation among more complex organisms.^7^^,^^60^^-^74 These findings suggested that miRNAs are likely part of an ancient regulatory mechanism. Recently, the majority of miRNA genes in human and chimpanzee embryonic stem cells involved in the regulation of pluripotency, self-renewal, and early decision of cell fate were mapped to chromosomes 19 and X.^75^ Interestingly, many of the miRNAs identified in this study that were of statistical significance values in hemangiomas were also mapped to chromosomes 19 and X (Table 1). This, parallel with embryonic stem cells, may provide insight into the origin, pathogenesis, and behavior of hemangiomas.

Microarray technology examining miRNA expression has yielded a substantial and growing amount of data about miRNAs, specifically supporting their role in tumor pathogenesis. While a single miRNA can play seemingly contradictory roles by regulating more than one gene or by changing activity based on the tumor microenvironment, certain miRNAs studied have been persistently linked to either tumor suppression or tumorigenesis. Therefore, the upregulation or downregulation of these miRNAs may provide valuable prognostic information in certain cancers by unveiling their malignant potential. In our study, a majority of the upregulated miRNAs in our hemangioma specimens were strongly linked to tumor suppression: miR-143,^76^ miR-145,^77^ miR-451,^78^ miR-515-5p,^79^ miR-30b,^80^ miR-15a,^81^ let-7g,^82^ miR-126,^83^ miR-512-3p,^84^ and miR-647.^85^ In addition, many of the downregulated miRNAs in our hemangioma samples were strongly linked to tumorigenesis: miR-423-5p,^86^ miR-93,^87^ miR-1180,^88^ miR-197,^89^ miR-299-5p,^90^ miR-224,^91^ miR-766,^92^ and miR-17.^93^ This balance between the expression of tumor-suppressive and oncogenic miRNAs may offer evidence as to why the scale is tipped toward “benign” for hemangiomas.

The expression profile of miRNAs in our hemangioma specimens shared similarities to that of published data on miRNA regulation of stem cells, particularly mesenchymal stem cells (MSCs) and neural crest cells. These multipotent cells are of particular interest because their potential to replicate as undifferentiated cells and their potential to differentiate into various cell lineages are in part regulated by miRNAs. The correlation of the miRNA expression profile of our hemangioma specimens to that of MSCs and neural crest cells can provide insight into the evolution of hemangiomas from these multipotent progenitor cells. A review by Clark et al^94^ highlighted a consensus list of miRNAs that were expressed in all MSCs (ie, from adipose tissue, bone marrow, umbilical cord). We found that the following miRNAs were also differentially expressed in our hemangioma specimens: let-7g, miR-23b, miR-29b-1, miR-143, miR-145, and miR-320a. This consensus list by no means paints the entire picture as a large number of other miRNAs have more specific roles in influencing MSC behavior. An example of this is found in miR-126, a miRNA that was significantly upregulated in our hemangioma specimens. In a study by Chen and Zhou,^95^ miR-126 promoted angiogenesis and improved cardiac function when transfected into MSCs and implanted onto the infarcted myocardium of mice. This study highlights how a particular miRNA regulates the paracrine effects of MSCs by promoting angiogenesis.^94^ Data on miRNAs and neural crest development are not as extensive. However, a review by Strobl-Mazzulla et al^96^ highlighted several miRNAs as regulators of neural crest cell behavior. miR-145, which was upregulated in our hemangioma specimens, is of particular interest because a study by Cordes et al^97^ showed that miR-145 was solely sufficient to induce differentiation of neural crest cells into vascular smooth muscle cells (VSMCs). Taking studies such as these into account, we can begin to understand how miRNAs influence the development of hemangiomas.

After the initial proliferative phase, hemangiomas enter into a quiescent or rest phase. Our hemangioma specimens exhibited significantly increased expression of several miRNAs involved in VSMC regulation (miR-143, miR-145, miR-195, and miR-424), which provides insight into this hemangioma process. Cordes et al^97^ showed that miR-143 and miR-145 promote a quiescent VSMC phenotype in already established VSMCs by inhibiting their proliferation and stabilizing their differentiated state. miR-195 has been shown to inhibit VSMC proliferation and migration through the downregulation of cell division cycle 42 (Cdc42).^98^ In addition, in a murine study by Merlet et al,^99^ miR-424 expression temporally increased after carotid artery injury; miR-424 overexpression repressed VSMC proliferation and de-differentiation by decreasing expression of cyclin D1, calumenin, and stromal-interacting molecule 1 (STIM1). Taken together, the upregulation of these miRNAs in our hemangioma specimens appears to modulate hemangioma proliferation and “stabilize” these tumors into their quiescent phase. Further evidence for our hypothesis has been recently described by Huang et al,^100^ who showed that miR-143 arrested proliferation of hemangioma-derived endothelial cells through the downregulation of B-cell lymphoma 2 (Bcl-2).

The eventual fibrofatty involution of hemangiomas indicates that adipocyte differentiation is likely involved in the pathogenesis of hemangiomas, and a large number of miRNAs have already been implicated in the regulation of adipocyte differentiation.^94^ We found significant differential expression of the following miRNAs in our own hemangioma specimens: miR-143, let-7g, miR-30b, miR-29b-1, and miR-17-5p. miR-143, let-7g, and miR-30b/e were significantly upregulated, and miR-29b-1 and miR-17-5p were significantly downregulated. miR-143 has been shown to suppress the expression of delta-like noncanonical Notch ligand 1 (DLK) in preadipocytes and promote adipocyte differentiation.^101^ DLK expression has been previously shown to be significantly increased in hemangiomas.^102^ More importantly, however, DLK expression decreases by at least 3-fold as hemangiomas progress from their proliferative phase to their involuting phase.^102^^,^^103^ DLK, synthesized as a transmembrane protein, is a powerful repressor of adipocyte differentiation.^104^ Therefore, its decrease, at least partly regulated by the increased expression of miR-143, is consistent with the adipocyte differentiation that occurs as hemangiomas involute. Likewise, the expression patterns of the other miRNAs listed earlier are also consistent with the process of fibrofatty involution in the literature.^105^^-^108

This study has the following limitations. Comparing selectively isolated endothelial cells from hemangiomas to normal cultured endothelial cells would have controlled for admixture and the potential changes induced by the cultured environment. Unfortunately, we were unable to do this for various technical and logistical reasons. Cultured HDMECs were used to obtain a large enough quantity of cells for the purpose of this experiment (miRNA microarray analysis). While other studies have used normal tissues as controls,^109^ we felt that this was not representative of a normal endothelial cell miRNA control. Data compared by hemangioma phase were not found to be statistically significant, which we attributed to inadequate sample size in each group (data not shown). In this era of widespread propranolol use, further sample procurement was deemed impractical. Nevertheless, we believe our data are a good first approximation of the results despite the aforementioned limitations. This is justified by the data's consistency with the biology and behavior of hemangiomas as we understand them in the literature.

The differential expression of miRNAs found in our hemangioma specimens provides insight into the character and evolution of hemangiomas as they proliferate, stabilize, and eventually involute. miRNAs involved in the paracrine effects of MSCs and neural crest cell differentiation such as miR-126 and miR-145 may regulate the formation and proliferation of hemangiomas. The miRNAs, miR-143, miR-145, miR-195, and miR-424, may stabilize these vascular tumors, whereas miR-143, let-7g, miR-30b/e, miR-29b-1, and miR-17-5p may influence their fibrofatty involution. Further investigation into the temporal relationship of the differential expression of miRNAs may provide further understanding into how these seemingly aggressive tumors from their onset are in fact benign, involuting lesions. For those lesions that necessitate intervention, therapeutic strategies involving miRNAs remain to be explored. While initially expected, piRNAs transcripts were not detected in the hemangioma specimens.

## Figures and Tables

**Table 1:** Statistically different expression miRNA signals between control microvascular endothelial cells and hemangioma tissue, P < 5.56E-5*

miRNA	Chromosome	Control mean signal	Hemangioma mean signal	*P*
Downregulated (N = 18)				
hsa-miR-423-5p	17	8,387	919	5.61E−07
hsa-miR-93	7	3,577	568	6.94E−07
hsa-miR-29b-1	7	242	49	7.75E−07
hsa-miR-320c	1	8,892	2,504	1.20E−06
hsa-miR-320a	18	9,216	2,654	2.65E−06
hsa-miR-584	5	4,655	42	2.90E−06
hsa-miR-1180	17	1040	46	2.90E−06
hsa-miR-320b	X	7,592	2,299	2.95E−06
hsa-miR-197	1	1,780	105	3.19E−06
hsa-miR-1238	19	129	41	4.26E−06
hsa-miR-299-5p	14	321	109	6.04E−06
hsa-miR-151-3p	8	1,807	577	1.08E−05
hsa-miR-224	X	2,295	230	1.31E−05
hsa-miR-485-3p	14	568	39	2.30E−05
hsa-miR-766	X	145	58	2.32E−05
hsa-miR-106a	X	2,411	1,087	2.48E−05
hsa-miR-320d	8	6,658	1,648	2.58E−05
hsa-miR-17	13	2,757	1,170	2.75E−05
Upregulated (N = 15)				
hsa-miR-143	5	141	15,013	3.40E−10
hsa-miR-145	5	375	18,275	2.05E−09
hsa-miR-195	17	1,256	14,854	4.11E−09
hsa-miR-451	19	206	13,351	6.27E−07
hsa-miR-152	17	417	1,559	1.48E−06
hsa-miR-515-5p	19	108	1,155	2.52E−06
hsa-miR-424	X	169	5,896	2.90E−06
hsa-miR-30b	8	1,432	8,207	2.90E−06
hsa-miR-15a	13	497	4,760	6.83E−06
hsa-let-7g	3	8,255	11,026	7.49E−06
hsa-miR-30e	1	625	3,008	8.60E−06
hsa-miR-23b	9	14,477	21,234	1.85E−05
hsa-miR-126	9	18,176	32,216	2.08E−05
hsa-miR-512-3p	19	101	513	2.27E−05
hsa-miR-647	20	7	22	5.05E−05

*A total of 18 downregulated and 15 upregulated miRNAs were statistically significant. miRNA indicates microRNA.

## Footnotes

* Performed in part as a service by LC Sciences, Houston, Tex.

## References

[1] Wijnhoven BP, Michael MZ, Watson DI (2007). MicroRNAs and cancer. Br J Surg.

[2] Ambros V (2004). The functions of animal microRNAs. Nature.

[3] Abbott AL, Alvarez-Saavedra E, Miska EA (2005). The let-7 microRNA family members mir-48, mir-84, and mir-241 function together to regulate developmental timing in *Caenorhabditis elegans*. Dev Cell.

[4] Seggerson K, Tang L, Moss EG (2002). Two genetic circuits repress the *Caenorhabditis elegans* heterochronic gene lin-28 after translation initiation. Dev Biol.

[5] Ambros V (2003). MicroRNA pathways in flies and worms: growth, death, fat, stress, and timing. Cell.

[6] Bartel DP (2004). MicroRNAs: genomics, biogenesis, mechanism, and function. Cell.

[7] Lai EC (2003). microRNAs: runts of the genome assert themselves. Curr Biol.

[8] Erdmann VA, Barciszewska MZ, Szymanski M (2001). The non-coding RNAs as riboregulators. Nucleic Acids Res.

[9] Lee RC, Feinbaum RL, Ambros V (1993). The C. elegans heterochronic gene lin-4 encodes small RNAs with antisense complementarity to lin-14. Cell.

[10] Reinhart BJ, Slack FJ, Basson M (2000). The 21-nucleotide let-7 RNA regulates developmental timing in *Caenorhabditis elegans*. Nature.

[11] Wightman B, Ha I, Ruvkun G (1993). Posttranscriptional regulation of the heterochronic gene lin-14 by lin-4 mediates temporal pattern formation in *C. elegans*. Cell.

[12] John B, Enright AJ, Aravin A (2004). Human MicroRNA targets. PLoS Biol.

[13] Xie X, Lu J, Kulbokas EJ (2005). Systematic discovery of regulatory motifs in human promoters and 3′ UTRs by comparison of several mammals. Nature.

[14] Calin GA, Dumitru CD, Shimizu M (2002). Frequent deletions and down-regulation of micro-RNA genes miR15 and miR16 at 13q14 in chronic lymphocytic leukemia. Proc Natl Acad Sci U S A.

[15] McManus MT (2003). MicroRNAs and cancer. Semin Cancer Biol.

[16] Cummins JM, Velculescu VE (2006). Implications of micro-RNA profiling for cancer diagnosis. Oncogene.

[17] Lu J, Getz G, Miska EA (2005). MicroRNA expression profiles classify human cancers. Nature.

[18] King HC, Sinha AA (2001). Gene expression profile analysis by DNA microarrays: promise and pitfalls. JAMA.

[19] Hoheisel JD (2006). Microarray technology: beyond transcript profiling and genotype analysis. Nat Rev Genet.

[20] Michael MZ, O’ Connor SM, van Holst Pellekaan NG (2003). Reduced accumulation of specific microRNAs in colorectal neoplasia. Mol Cancer Res.

[21] Calin GA, Liu CG, Sevignani C (2004). MicroRNA profiling reveals distinct signatures in B cell chronic lymphocytic leukemias. Proc Natl Acad Sci U S A.

[22] Metzler M, Wilda M, Busch K (2004). High expression of precursor microRNA-155/BIC RNA in children with Burkitt lymphoma. Genes Chromosomes Cancer.

[23] Kluiver J, Haralambieva E, de Jong D (2006). Lack of BIC and microRNA miR-155 expression in primary cases of Burkitt lymphoma. Genes Chromosomes Cancer.

[24] Eis PS, Tam W, Sun L (2005). Accumulation of miR-155 and BIC RNA in human B cell lymphomas. Proc Natl Acad Sci U S A.

[25] Kluiver J, Poppema S, de Jong D (2005). BIC and miR-155 are highly expressed in Hodgkin, primary mediastinal and diffuse large B cell lymphomas. J Pathol.

[26] Costinean S, Zanesi N, Pekarsky Y (2006). Pre-B cell proliferation and lymphoblastic leukemia/high-grade lymphoma in E(mu)-miR155 transgenic mice. Proc Natl Acad Sci U S A.

[27] Akao Y, Nakagawa Y, Kitade Y (2007). Downregulation of microRNAs-143 and -145 in B-cell malignancies. Cancer Sci.

[28] Bottoni A, Piccin D, Tagliati F (2005). miR-15a and miR-16-1 down-regulation in pituitary adenomas. J Cell Physiol.

[29] Amaral FC, Torres N, Saggioro F (2009). MicroRNAs differentially expressed in ACTH-secreting pituitary tumors. J Clin Endocrinol Metab.

[30] Takamizawa J, Konishi H, Yanagisawa K (2004). Reduced expression of the let-7 microRNAs in human lung cancers in association with shortened postoperative survival. Cancer Res.

[31] Johnson SM, Grosshans H, Shingara J (2005). RAS is regulated by the let-7 microRNA family. Cell.

[32] Huang TH, Wu F, Loeb GB (2009). Up-regulation of miR-21 by HER2/neu signaling promotes cell invasion. J Biol Chem.

[33] Kato M, Paranjape T, Müller RU (2009). The mir-34 microRNA is required for the DNA damage response in vivo in *C. elegans* and in vitro in human breast cancer cells. Oncogene.

[34] Khoshnaw SM, Green AR, Powe DG (2009). MicroRNA involvement in the pathogenesis and management of breast cancer. J Clin Pathol.

[35] Iorio MV, Ferracin M, Liu CG (2005). MicroRNA gene expression deregulation in human breast cancer. Cancer Res.

[36] Hu X, Macdonald DM, Huettner PC (2009). A miR-200 microRNA cluster as prognostic marker in advanced ovarian cancer. Gynecol Oncol.

[37] Iorio MV, Visone R, Di Leva G (2007). MicroRNA signatures in human ovarian cancer. Cancer Res.

[38] Cui JG, Zhao Y, Sethi P (2010). Micro-RNA-128 (miRNA-128) down-regulation in glioblastoma targets ARP5 (ANGPTL6), Bmi-1 and E2F-3a, key regulators of brain cell proliferation. J Neurooncol.

[39] Silber J, James CD, Hodgson JG (2009). microRNAs in gliomas: small regulators of a big problem. Neuromolecular Med.

[40] Chan JA, Krichevsky AM, Kosik KS (2005). MicroRNA-21 is an antiapoptotic factor in human glioblastoma cells. Cancer Res.

[41] Ciafrè SA, Galardi S, Mangiola A (2005). Extensive modulation of a set of microRNAs in primary glioblastoma. Biochem Biophys Res Commun.

[42] He H, Jazdzewski K, Li W (2005). The role of microRNA genes in papillary thyroid carcinoma. Proc Natl Acad Sci U S A.

[43] Yamamoto Y, Kosaka N, Tanaka M (2009). MicroRNA-500 as a potential diagnostic marker for hepatocellular carcinoma. Biomarkers.

[44] Murakami Y, Yasuda T, Saigo K (2006). Comprehensive analysis of microRNA expression patterns in hepatocellular carcinoma and non-tumorous tissues. Oncogene.

[45] Kim VN (2006). Small RNAs just got bigger: Piwi-interacting RNAs (piRNAs) in mammalian testes. Genes Dev.

[46] Vagin VV, Sigova A, Li C (2006). A distinct small RNA pathway silences selfish genetic elements in the germline. Science.

[47] Saito K, Nishida KM, Mori T (2006). Specific association of Piwi with rasiRNAs derived from retrotransposon and heterochromatic regions in the *Drosophila* genome. Genes Dev.

[48] Gunawardane LS, Saito K, Nishida KM (2007). A slicer-mediated mechanism for repeat-associated siRNA 5′ end formation in *Drosophila*. Science.

[49] Pal-Bhadra M, Leibovitch BA, Gandhi SG (2004). Heterochromatic silencing and HP1 localization in *Drosophila* are dependent on the RNAi machinery. Science.

[50] Kalmykova AI, Klenov MS, Gvozdev VA (2005). Argonaute protein PIWI controls mobilization of retrotransposons in the *Drosophila* male germline. Nucleic Acids Res.

[51] Smith CJF, Friedlander SF, Guma M (2017). Infantile hemangiomas: an updated review on risk factors, pathogenesis, and treatment. Birth Defects Res.

[52] Chen TS, Eichenfield LF, Friedlander SF (2013). Infantile hemangiomas: an update on pathogenesis and therapy. Pediatrics.

[53] Holland KE, Drolet BA (2010). Infantile hemangioma. Pediatr Clin North Am.

[54] Liang MG, Frieden IJ (2014). Infantile and congenital hemangiomas. Semin Pediatr Surg.

[55] Bowers RE, Graham EA, Tomlinson KM (1960). The natural history of the strawberry nevus. Arch Dermatol.

[56] Mulliken JB, Glowacki J (1982). Hemangiomas and vascular malformations in infants and children: a classification based on endothelial characteristics. Plast Reconstr Surg.

[57] Enjolras O, Mulliken JB (1997). Vascular tumors and vascular malformations (new issues). Adv Dermatol.

[58] Bruckner AL, Frieden IJ (2003). Hemangiomas of infancy. J Am Acad Dermatol.

[59] Pasquinelli AE, Ruvkun G (2002). Control of developmental timing by microRNAs and their targets. Annu Rev Cell Dev Biol.

[60] Lagos-Quintana M, Rauhut R, Lendeckel W (2001). Identification of novel genes coding for small expressed RNAs. Science.

[61] Lagos-Quintana M, Rauhut R, Yalcin A (2002). Identification of tissue-specific microRNAs from mouse. Curr Biol.

[62] Lau NC, Lim LP, Weinstein EG (2001). An abundant class of tiny RNAs with probable regulatory roles in *Caenorhabditis elegans*. Science.

[63] Lee RC, Ambros V (2001). An extensive class of small RNAs in *Caenorhabditis elegans*. Science.

[64] Pasquinelli AE, Reinhart BJ, Slack F (2000). Conservation of the sequence and temporal expression of let-7 heterochronic regulatory RNA. Nature.

[65] Ambros V, Lee RC, Lavanway A (2003). MicroRNAs and other tiny endogenous RNAs in *C. elegans*. Curr Biol.

[66] Aravin AA, Naumova NM, Tulin AV (2001). Double-stranded RNA-mediated silencing of genomic tandem repeats and transposable elements in the *D. melanogaster* germline. Curr Biol.

[67] Lagos-Quintana M, Rauhut R, Meyer J (2003). New microRNAs from mouse and human. RNA.

[68] Dostie J, Mourelatos Z, Yang M (2003). Numerous microRNPs in neuronal cells containing novel microRNAs [published correction appears in: *RNA*. 2003;9(5):631-2]. RNA.

[69] Houbaviy HB, Murray MF, Sharp PA (2003). Embryonic stem cell-specific microRNAs. Dev Cell.

[70] Krichevsky AM, King KS, Donahue CP (2003). A microRNA array reveals extensive regulation of microRNAs during brain development. RNA.

[71] Petruzzo P, Revillard JP, Kanitakis J (2003). First human double hand transplantation: efficacy of a conventional immunosuppressive protocol. Clin Transplant.

[72] Lim LP, Lau NC, Weinstein EG (2003). The microRNAs of *Caenorhabditis elegans*. Genes Dev.

[73] Lim LP, Glasner ME, Yekta S (2003). Vertebrate microRNA genes. Science.

[74] Moss EG, Tang L (2003). Conservation of the heterochronic regulator Lin-28, its developmental expression and microRNA complementary sites. Dev Biol.

[75] Cao H, Yang CS, Rana TM (2008). Evolutionary emergence of microRNAs in human embryonic stem cells. PLoS One.

[76] Ma Z, Luo Y, Qiu M (2017). miR-143 induces the apoptosis of prostate cancer LNCap cells by suppressing Bcl-1 expression. Med Sci Monit.

[77] Zhou X, Yue Y, Wang R (2017). MicroRNA-145 induces tumorigenesis and invasion of cervical cancer stem cells. Int J Oncol.

[78] Goto A, Tanaka M, Yoshida M (2017). The low expression of miR-451 predicts a worse prognosis in non-small cell lung cancer cases. PLoS One.

[79] Pardo OE, Castellano L, Munro CE (2016). miR-515-5p controls cancer cell migration through MARK4 regulation. EMBO Rep.

[80] Li Q, Zhang X, Li N (2017). miR-30b inhibits cancer cell growth, migration, and invasion by targeting homeobox A1 in esophageal cancer. Biochem Biophys Res Commun.

[81] Zhu K, He Y, Xia C (2016). MicroRNA-15a inhibits proliferation and induces apoptosis in CNE1 nasopharyngeal carcinoma cells. Oncol Res.

[82] Hu H, Zhao X, Jin Z (2015). Hsa-let-7g miRNA regulates the anti-tumor effects of gastric cancer cells under oxidative stress through the expression of DDR genes. J Toxicol Sci.

[83] Ebrahimi F, Gopalan V, Wahab R (2015). Deregulation of miR-126 expression in colorectal cancer pathogenesis and its clinical significance. Exp Cell Res.

[84] Zhu X, Gao G, Chu K (2015). Inhibition of RAC1-GEF DOCK3 by miR-512-3p contributes to suppression of metastasis in non-small cell lung cancer. Int J Biochem Cell Biol.

[85] Cao W, Wei W, Zhan Z (2017). Role of miR-647 in human gastric cancer suppression. Oncol Rep.

[86] Li S, Zeng A, Hu Q (2017). miR-423-5p contributes to a malignant phenotype and temozolomide chemoresistance in glioblastomas. Neuro Oncol.

[87] Liang H, Wang F, Chu D (2016). miR-93 functions as an oncomiR for the downregulation of PDCD4 in gastric carcinoma. Sci Rep.

[88] Zhou X, Zhu HQ, Ma CQ (2016). miR-1180 promoted the proliferation of hepatocellular carcinoma cells by repressing TNIP2 expression. Biomed Pharmacother.

[89] Remon J, Alvarez-Berdugo D, Majem M (2016). miRNA-197 and miRNA-184 are associated with brain metastasis in EGFR-mutant lung cancers. Clin Transl Oncol.

[90] Wu SQ, Zhang LH, Huang HB (2016). miR-299-5p promotes cell growth and regulates G1/S transition by targeting p21Cip1/Waf1 in acute promyelocytic leukemia. Oncol Lett.

[91] Cui R, Meng W, Sun HL (2015). MicroRNA-224 promotes tumor progression in nonsmall cell lung cancer. Proc Natl Acad Sci U S A.

[92] Li YC, Li CF, Chen LB (2015). MicroRNA-766 targeting regulation of SOX6 expression promoted cell proliferation of human colorectal cancer. Onco Targets Ther.

[93] Xi XP, Zhuang J, Teng MJ (2016). MicroRNA-17 induces epithelial-mesenchymal transition consistent with the cancer stem cell phenotype by regulating CP7B1 expression in colon cancer. Int J Mol Med.

[94] Clark EA, Kalomoiris S, Nolta JA (2014). Concise review: micro RNA function in multipotent mesenchymal stromal cells. Stem Cells.

[95] Chen JJ, Zhou SH (2011). Mesenchymal stem cells overexpressing miR-126 enhance ischemic angiogenesis via the AKT/ERK-related pathway. Cardiol J.

[96] Strobl-Mazzulla PH, Marini M, Buzzi A (2012). Epigenetic landscape and miRNA involvement during neural crest development. Dev Dyn.

[97] Cordes KR, Sheehy NT, White MP (2009). miR-145 and miR-143 regulate smooth muscle cell fate and plasticity. Nature.

[98] Wang YS, Wang HY, Liao YC (2012). MicroRNA-195 regulates vascular smooth muscle cell phenotype and prevents neointimal formation. Cardiovasc Res.

[99] Merlet E, Atassi F, Motiani RK (2013). miR-424/322 regulates vascular smooth muscle cell phenotype and neointimal formation in the rat. Cardiovasc Res.

[100] Huang C, Huang J, Ma P (2017). microRNA-143 acts as a suppressor of hemangioma growth by targeting Bcl-2. Gene.

[101] Kim YJ, Min TS, Seo KS (2015). Expression of pref-1/dlk-1 is regulated by microRNA-143 in 3T3-L1 cells. Mol Biol Rep.

[102] Spock CL, Tom LK, Canadas K (2015). Infantile hemangiomas exhibit neural crest and pericyte markers. Ann Plast Surg.

[103] Ritter MR, Dorrell MI, Edmonds J (2002). Insulin-like growth factor 2 and potential regulators of hemangioma growth and involution identified by large-scale expression analysis. Proc Natl Acad Sci U S A.

[104] Smas CM, Sul HS (1993). Pref-1, a protein containing EGF-like repeats, inhibits adipocyte differentiation. Cell.

[105] Sun T, Fu M, Bookout AL (2009). MicroRNA let-7 regulates 3T3-L1 adipogenesis. Mol Endocrinol.

[106] Zaragosi LE, Wdziekonski B, Brigand KL (2011). Small RNA sequencing reveals miR-642a-3p as a novel adipocyte-specific microRNA and miR-30 as a key regulator of human adipogenesis. Genome Biol.

[107] Zhu Y, Zheng G, Wang H (2017). Downregulated miR-29a/b/c during contact inhibition stage promote 3T3-L1 adipogenesis by targeting DNMT3A. PLoS One.

[108] Liu Y, Liu W, Hu C (2011). MiR-17 modulates osteogenic differentiation through a coherent feed-forward loop in mesenchymal stem cells isolated from periodontal ligaments of patients with periodontitis. Stem Cells.

[109] Nakashima T, Jinnin M, Etoh T (2010). Down-regulation of mir-424 contributes to the abnormal angiogenesis via MEK1 and cyclin E1 in senile hemangioma: its implications to therapy. PLoS One.

